# Molecular Imaging Findings on Acute and Long-Term Effects of COVID-19 on the Brain: A Systematic Review

**DOI:** 10.2967/jnumed.121.263085

**Published:** 2022-07

**Authors:** Philipp T. Meyer, Sabine Hellwig, Ganna Blazhenets, Jonas A. Hosp

**Affiliations:** 1 Department of Nuclear Medicine, Medical Center - University of Freiburg and Faculty of Medicine, University of Freiburg, Freiburg, Germany;; 2 Department of Psychiatry and Psychotherapy, Medical Center - University of Freiburg and Faculty of Medicine, University of Freiburg, Freiburg, Germany; and; 3 Department of Neurology and Clinical Neuroscience, Medical Center - University of Freiburg and Faculty of Medicine, University of Freiburg, Freiburg, Germany

**Keywords:** neurology, PET, SPECT, COVID-19, SARS-CoV-2, SPECT, brain

## Abstract

Molecular imaging techniques such as PET and SPECT have been used to shed light on how coronavirus disease 2019 (COVID-19) affects the human brain. We provide a systematic review that summarizes the current literature according to 5 predominant topics. First, a few case reports have suggested reversible cortical and subcortical metabolic alterations in rare cases with concomitant para- or postinfectious encephalitis. Second, imaging findings in single patients with the first manifestations of parkinsonism in the context of COVID-19 resemble those in neurodegenerative parkinsonism (loss of nigrostriatal integrity), but scarceness of data and a lack of follow-up preclude further etiologic conclusions (e.g., unmasking/hastening of neurodegeneration vs. infectious or parainfectious parkinsonism). Third, several case reports and a few systematic studies have addressed focal symptoms and lesions, most notably hyposmia. The results have been variable, although some studies found regional hypometabolism of regions related to olfaction (e.g., orbitofrontal and mesiotemporal). Fourth, a case series and systematic studies in inpatients with COVID-19–related encephalopathy (acute to subacute stage) consistently found a frontoparietal-dominant neocortical dysfunction (on imaging and clinically) that proved to be grossly reversible in most cases until 6 mo. Fifth, studies on post–COVID-19 syndrome have provided controversial results. In patients with a high level of self-reported complaints (e.g., fatigue, memory impairment, hyposmia, and dyspnea), some authors found extensive areas of limbic and subcortical hypometabolism, whereas others found no metabolic alterations on PET and only minor cognitive impairments (if any) on neuropsychologic assessment. Furthermore, we provide a critical appraisal of studies with regard to frequent methodologic issues and current pathophysiologic concepts. Finally, we devised possible applications of PET and SPECT in the clinical work-up of diagnostic questions related to COVID-19.

Neurologic complications (1) and neurocognitive aftermaths (2) are frequently described in coronavirus disease 2019 (COVID-19), which is caused by an infection with the severe acute respiratory syndrome coronavirus 2 (SARS-CoV-2). Neurologic symptoms are present in roughly two thirds of hospitalized COVID-19 patients, being associated with a more severe course of disease (3), higher age, and preexisting comorbidities (4). So far, the most widely accepted classification of COVID-19–related symptoms refers to their timing with respect to symptom onset (5): the acute phase covers the first 4 wk, whereas the terminus ongoing symptomatic COVID-19 designates the period between the fifth and twelfth weeks after symptom onset. Symptoms that persist for longer than 12 wk and are not explained by an alternative diagnosis are finally subsumed as a post–COVID-19 syndrome (also referred to as long–COVID-19 syndrome).

During the acute phase of the disease, the following complications have been reported with decreasing frequency: disturbance of smell or taste in approximately 60%–80% of patients (6), myalgia as a sign of viral myositis in approximately 40% of patients (7), encephalopathies with various manifestations in 15%–30% of hospitalized patients (3*,*^8^*,*^9^), cerebrovascular events (e.g., stroke, hemorrhage, or sinus vein thrombosis) in 1%–5% of hospitalized patients (10), peripheral neuropathies ranging from cranial nerve palsies to Guillain–Barré syndrome in larger case series (11*,*^12^), and, only occasionally, encephalitic manifestations (13).

Regarding the phase of ongoing symptomatic COVID-19, various studies report lasting neuropsychiatric symptoms such as fatigue, memory loss, and attentional problems (14*,*^15^). Furthermore, cognitive impairment affecting attentional and executive functions, memory, and visuoconstruction are ascertainable by neuropsychologic test batteries (16–18). Especially in patients treated as inpatients or with more severe clinical courses, some of these symptoms may represent residual, though recovering, deficits from an initial COVID-19–related encephalopathy.

Among other symptoms, fatigue, subjective cognitive impairment, and headache extend into the phase of post–COVID-19 syndrome (2*,*^19^). Although the term *post–COVID-19 syndrome* in its strict sense (5) is detached from particular symptoms, it is widely used as a label for a syndrome enveloping lasting fatigue, cognitive problems, and shortness of breath, which affect approximately 10% of all patients (2*,*^19^). These symptoms, unlike symptoms in cases with COVID-19–related encephalopathy, do not necessarily occur with or shortly after infection but may be noticed weeks to even months later and affect not only initially severely affected patients but also those with an uncomplicated, ambulatory disease course (2*,*^19^). During the preparation of this review, the World Health Organization (WHO) published a definition of “post–COVID-19 condition” that demands, in addition to a period of usually 3 mo from symptom onset, the presence of at least one specified symptom (e.g., fatigue or cognitive dysfunction) lasting for at least 2 mo and exerting a relevant impact on everyday functioning (20). Because of the novelty of this publication, all reports reviewed here used institutional or earlier definitions (5).

Furthermore, the incidence of dementia, parkinsonism, and psychiatric disorders (i.e., mood, anxiety, and psychosis) within 6 mo after infection is significantly higher in patients who had COVID-19 than in those affected by influenza or other respiratory tract infections (21). Here, encephalopathy during the acute phase turned out to be the most important risk factor. Thus, COVID-19 may unmask subclinical neurodegenerative disorders or worsen preexisting conditions.

Molecular imaging techniques such as PET and SPECT have been used for the diagnostic work-up of neurologic COVID-19 manifestations. These examinations provided a plethora of sometimes conflicting results in highly variable populations that are often ill defined in terms of symptoms and temporal course. Thus, the objective of the present systematic review is to provide a comprehensive, structured, and critical survey of actual knowledge on molecular imaging in neuropsychiatric COVID-19 manifestations. In addition, we provide a preliminary suggestion on possible future use of PET and SPECT in this particular field of application based on the literature and our personal experience.

## MATERIALS AND METHODS

We conducted a MEDLINE (https://www.ncbi.nlm.nih.gov) literature search to identify peer-reviewed original studies and case series or case reports using PET or SPECT to investigate central nervous system (CNS) manifestations of COVID-19. The following search criteria were applied: “(corona OR COVID OR SARS-CoV-2) AND (PET OR positron OR SPECT OR single-photon) AND (brain OR cerebral),” limited to 2019–2021. Titles, abstracts, full-text articles, and references were screened to identify appropriate reports. In addition, we conducted a selective search for current literature on general, neurologic, and psychiatric aspects of COVID-19 and related topics to embed the imaging finding into clinical and scientific context.

## INSIGHTS FROM MOLECULAR IMAGING WITH PET AND SPECT

The literature search yielded 53 hits (as of November 1, 2021), of which 25 publications included the results of PET and SPECT examinations in patients with various neurologic symptoms and complaints at variable time points after an infection with SARS-CoV-2. These reports comprise 15 case reports or case series (Supplemental Table 1; supplemental materials are available at http://jnm.snmjournals.org) and 10 original publications (Tables 1 and 2; Supplemental Table 2). For the sake of clarity, we assigned all reports to 1 of the following 5 topics. According to current knowledge, this assignment does not imply that different mechanisms necessarily underlie each different topic or that these are mutually exclusive.

**TABLE 1:** Systematic Studies on Molecular Imaging in Cerebral Manifestations of COVID-19: Encephalopathy

Parameter	Kas et al. (39)	Hosp et al. (18)	Blazhenets et al. (41)
Research question	Longitudinal metabolic pattern in COVID-19 encephalopathy	Neuronal correlates of neurologic and cognitive symptoms (subacute stage)	Recovery of cognitive impairment and regional hypometabolism in subacute COVID-19 during 5- to 6-mo follow-up
Population			
Inclusion (main)	PCR+; new cognitive impairment with focal CNS sign or seizure; other infectious or autoimmune disorders excluded	PCR+; only inpatients; ≥1 new neurologic symptom (including cognition [MoCA]); PET, MRI, and neuropsychologic test battery if ≥2 new symptoms	Follow-up data of Hosp et al. (18); clinical register, 17 pts with PET during subacute phase; 9 pts without complaints refused follow-up PET
* n*	7	15 (PET)	8
Age (y)	50–72	65 ± 14	66 ± 14
Selected clinical findings	All hospitalized (7); ventilated (3); executive deficit, frontal lobe changes (7); psychiatric manifestation (5); follow-up: improved (6) but residual attention/executive deficit at 4–8 mo; anxiety/depression (4); deterioration (1)	Initial ICU (7/29; 2 only observation; 2 noninvasive and 2 invasive ventilation); impaired gustation (29/29) and smell (25/29); impaired cognition (MoCA < 26; 18/26); prominent deficits in memory (7/14), executive functions (6/13), and attention (6/15); MRI mircoembolic infarcts (4/13); CSF: PCR− (4/29)	All treated as inpatients during acute phase, ICU (2); self-reported persistent cognitive deficits (4); MoCA: recovery from 19 ± 5 (1st) to 2 3± 4 (2nd examination), still impaired (5; especially memory)
^18^F-FDG PET			
Analysis	ROI; SPM: single-subject and group; normalization: scaling to pons; comparison: 32 NCs (identical protocol); *P* < 0.05 FWE	Single-case: visual inspection; PCA (scaled subprofile model); comparison: 45 control patients (identical protocol); plasma glucose–adjusted SUV; confirmatory analyses with SPM (normalization: white matter, *P* < 0.01 FDR) and PCA with 35 NC (similar scanner)	PCA: expression of previously established COVID-19–related covariance pattern; SPM: paired (within pts) and unpaired (vs. control patients); (Hosp et al. (18))
Δt	Acute, 4 wk later, or 26 wk after onset	4 ± 2 wk	23 ± 7 wk
Major findings	Acute—DEC: frontal, insula, cingulate, CN (group), and posterior cortices (6/7); INC: vermis, dentate nucleus, pons (group; *P* < 0.05, uncorrected); 4 wk later—DEC: frontal but improved (group); 26 wk after onset—almost normal, residual DEC orbitofrontal, insula, cingulate, CN (group); almost normal (3/7), frontal (3/7), improve/decline (1/7)	Single-case: predominant frontoparietal cortical DEC (10/15), relative INC of striatum (3/15) and vermis (1/15); group PCA: negative voxel weights (DEC) in extensive neocortical regions (especially frontoparietal) and CN; positive voxel weights (interpreted as preserved metabolism) in brain stem, CBL, MTL, and white matter; confirmatory analyses: similar results; significant negative correlation (*r*^2 ^= 0.62) MoCA vs. pattern expression score	PCA: pattern expression decreased (*P* = 0.002) but still at trend level higher than in control patients (*P* = 0.06); negative correlation (R^2 ^= 0.39) between MoCA and pattern expression; SPM (paired): significant recovery of neocortical DEC (*P* < 0.01 FDR); SPM (unpaired): residual neocortical DEC (trend level, *P* < 0.005, uncorrected)
Hypothesis	Widespread, frontal-dominant impairment, variably reversible in most patients until 6 mo, due to para- or postinfectious immune mechanism	Cortical dysfunction due to inflammatory process trigged by systemic immune response (e.g., cytokine release), particularly affecting white matter and being possibly reversible	Slow, but evident reversibility of lasting cortical dysfunction due to subcortical perinflammatory processes (triggered by systemic inflammatory response or cytokine release)

MoCA = Montreal Cognitive Assessment; pts = patients; ICU = intensive care unit; CSF = cerebrospinal fluid; PCR = polymerase chain reaction; ROI = region of interest; SPM = statistical parametric mapping; SPM: single-subject or group = SPM-group or single-subject analyses (usually COVID patients vs. NCs); normalization = method/reference region used for count rate normalization of PET scans; NCs = healthy controls; PCA = principal-components analysis; FDR = false-discovery rate correction; Δt = interval between symptom onset or PCR+ for SARS-CoV-2 (as available) and PET; DEC and INC = decreased and increased signal, respectively; CN = caudate nucleus; CBL = cerebellum; MTL = mesial temporal lobe.

Numbers in parentheses refer to number of subjects with specified finding (if subsample assessed is smaller than study group, sample size as indicated). ^18^F-FDG target parameter is glucose metabolism.

**TABLE 2:** Systematic Studies on Molecular Imaging in Cerebral Manifestations of COVID-19: Post–COVID-19 Syndrome

Parameter	Guedj et al. (43)	Sollini et al. (45)	Morand et al. (44)	Dressing et al. (46)
Research question	Metabolic pattern of long COVID	Whole-body PET/CT (including brain) to gain insights into long COVID (for whole body, see report)	Regional metabolic pattern in pediatric patients with suspected long COVID	Regional metabolic pattern in patients with neurocognitive long COVID
Population				
Inclusion (main)	Retrospective; >3 wk after SARS-CoV-2 infection (PCR+ or antibody-positive); persistent fatigue; PET for neurologic complaints; normal CT/MRI	Observational case-control study; ≥1 persistent symptom for >30 d after infection (PCR: NA); NCs: age- and sex-matched, surgically treated melanoma pts with negative PET/CT	Retrospective; children with suspected long COVID (clinical diagnosis); evaluation for various functional complaints ≥ 4 wk after suspected SARS-CoV-2 infection	History of PCR+ SARS-CoV-2 infection; new subjective neurocognitive symptoms > 3 mo since PCR+; no preexisting neurodegenerative disease; PET recommended to all pts (performed in 14/31; clinical findings in PET subgroup not different)
* n*	35	13	7	31
Age (y)	55 ± 11	54 (46–80)	12 (10–13)	14 (PET), 56 ± 7
Selected clinical findings	Hospitalized in ICU (12/31); ventilated (5/31); memory/cognitive complaints (17), insomnia (16), hyposmia (10)	Hospitalization (7/13); ventilated (2/13); dyspnea (9), fatigue (8), anosmia (4), ageusia (3)	Initial symptoms: fever (6), muscle pain (6), asthenia (5), rhinitis (5), hyposmia (5); long COVID symptoms: fatigue (5), cognitive impairment (5), dyspnea (4), headache (4); PCR+ (1/5) and positive SARS-CoV-2 serology (2/6)	Acute-phase inpatients (10; ICU 4); no current focal sign; subjective difficulties in attention and memory (31), fatigue (24), reduced work quota/unable to work (12); extensive neuropsychology: normal on group level, unimpaired test battery (15), but individual pts with deficits in memory domain (7/31) or impaired MoCA (9/31), fatigue (19/31)
^18^F-FDG PET				
Analysis	SPM: group; normalization: proportional scaling; comparison: 44 NCs (earlier study); *P* < 0.001, clusterwise *P* < 0.05 FWE	Brain PET extracted from whole-body scans; SPM: group; normalization: proportional scaling (global); comparison: 26 control patients; *P* < 0.001/0.005 (uncorrected)	SPM: group; normalization: proportional scaling (global); comparison: 21 pediatric control patients.; findings in adults (Guedj et al. (43)); *P* < 0.001, clusterwise *P* < 0.05 FWE	Single case: visual inspection plus single-case statistical analyses (3D-SSP); PCA: expression of COVID-19–related covariance pattern; SPM (confirmatory): group, normalization: brain parenchyma, *P* < 0.05 FDR; comparison: 45 control patients (Hosp et al., (18))
Δt (wk)	14 ± 4 (4–22)	19 ± 4	20 (4–34)	28 ± 9
Major findings	DEC: rectal/orbital gyrus, R temporal lobe (incl. MTL), R thalamus, pons/medulla, CBL; various weak correlations (*r*^2^ < 0.35), e.g., complaints (*n*), hyposmia, memory/cognitive complaint vs. CBL DEC	DEC (group contrast, *P* < 0.001)*: R parahippocampal, R thalamus; DEC in persistent anosmia/ageusia (*P* < 0.005)*: bilateral parahippocampal and orbitofrontal; DEC in persistent fatigue (*P* < 0.005)*: R parahippocampal, brain stem, bilateral thalamus	Comparison to pediatric control patients: bilateral DEC in MTL, pons, CBL; comparison to adult long COVID pts: no difference	Single case: no distinct pathologic finding; PCA: no elevated expression of COVID-19–related covariance pattern, no correlation with MoCA; SPM (confirmatory): no region of significantly different metabolism, no correlation with clinical scores
Hypothesis	SARS-CoV-2 neurotropism through olfactory bulb, extension of impairment to limbic or paralimbic regions, thalamus, CBL, and brain stem	Neuronal/synaptic dysfunction occurring after inflammatory changes triggered by SARS-CoV-2 infection	Several possible explanations (inflammatory, immune, neurotropism, vascular, gut–brain disturbance, psychologic), but none clearly favored	Factors other than those causing subacute cortical dysfunction in COVID-19 cause or contribute to symptoms in long COVID, in particular fatigue

* Questionable anatomic localization, hard to differentiate from CSF spaces.

3D-SSP = three-dimensional stereotactic surface projection; PCR = polymerase chain reaction; NA = not applicable; NCs = healthy controls; pts = patients; ICU = intensive care unit; DEC = decreased signal; MTL = mesial temporal lobe; CBL = cerebellum; PCA = principal-components analysis; MoCA = Montreal Cognitive Assessment.

Numbers in parentheses refer to number of subjects with specified finding (if subsample assessed is smaller than study group, sample size as indicated). ^18^F-FDG target parameter is glucose metabolism.

### Encephalitis

There were 3 independent case reports including ^18^F-FDG PET in patients with autoimmune encephalitis in the acute to subacute phase of COVID-19 (22–24). Given the preliminary nature of case reports, we are summarizing only the most noteworthy findings and refer the interested reader to Supplemental Table 1 for more detail. PET showed diffuse cortical hypometabolism (*n* = 1) and increased metabolism in the basal ganglia (*n* = 3), mesiotemporal structures (*n* = 1), and cerebellum (*n* = 1), possibly rated to cerebellum- and basal ganglia–specific antineuronal antibodies in 2 of these patients (22*,*^24^). Interestingly, immunomodulating treatment led to improvement in all patients, with 6-mo follow-up ^18^F-FDG PET having normal findings in 1 patient (24). The authors postulated para- or postinfectious encephalitis without evidence of direct virus invasion, which fits into other relative rare cases in the literature (13).

### Parkinsonism and Other Neurodegenerative Diseases

Three case reports described molecular imaging findings in 4 patients in whom the first manifestations of parkinsonism occurred only weeks after SARS-CoV-2 infection (in part with other neurologic symptoms such as myoclonus, fluctuating consciousness, ocular abnormalities, and cognitive impairment; Supplemental Table 1). Again, these findings have to be regarded as preliminary although raising the important question of whether and how a COVID-19–related CNS pathology may unmask or worsen neurodegeneration (21): imaging of dopamine transporter availability with ^123^I-FPCIT SPECT (25*,*^26^) and dopamine synthesis and storage with ^18^F-FDOPA PET (27) in 3 of these patients confirmed a nigrostriatal dopaminergic deficit. In 1 patient, normal cardiac innervation as assessed by ^123^I-MIBG scintigraphy and subsequent improvement without specific treatment argued against incipient Parkinson disease. The authors speculated that SARS-CoV-2 virus may have gained access to the CNS and affected the midbrain (25). Two patients showed clinical and ^18^F-FDG PET findings compatible with postinfectious immune-mediated encephalitis that bore similarities to the encephalitis lethargica that was seen after the influenza epidemic in 1918 (*26*). However, unlike encephalitis cases described in the paragraph above, these patients did not improve (neither spontaneously, nor with immunomodulatory treatment), and it is unclear whether parkinsonism might have been caused by subsequent development or unmasking and hastening of preclinical neurodegeneration (26). Similarly, a patient reported by Cohen et al. (27) was diagnosed with probable Parkinson disease according to current diagnostic criteria, possibly facilitated by genetic susceptibility or virus-induced inflammation. To the best of our knowledge, no imaging follow-up has yet been published, which would help to unravel the underlying mechanism. Finally, the possibility that SARS-CoV-2 infection may precipitate and accelerate neurodegenerative diseases was also raised by Young et al. (28), who presented the case of a previously healthy man who developed rapidly progressive and fatal Creutzfeldt–Jakob disease 2 wk after being diagnosed with COVID-19. In line with Creutzfeldt–Jakob disease, ^18^F-FDG PET showed left hemispheric hypometabolism. The authors referred to animal and human studies showing that proinflammatory cytokines may promote neuroinflammation and progression of various neurodegenerative forms of dementia and parkinsonism (28).

### Focal Symptoms or Lesions

Olfactory dysfunction is the most frequent focal neurologic sign in COVID-19. Several case reports (Supplemental Table 1) and 2 systematic studies (Supplemental Table 2) addressed this symptom. Niesen et al. (29) prospectively examined 12 patients with ^18^F-FDG PET/MRI about 2 wk after a sudden loss of smell in COVID-19. Single-subject and group analyses of ^18^F-FDG PET data showed no significant regional findings when using a conservative statistical threshold (i.e., *P* < 0.05, familywise error [FWE]–corrected). When using a liberal threshold (*P* < 0.001), individual analyses showed a heterogeneous pattern of metabolic decreases (*n* = 3), increases (*n* = 1), or both (*n* = 8) in various regions (e.g., olfactory regions, primary and higher-order cortices, and cerebellum), whereas group analyses resulted in clusters of hypometabolism in medial and dorsal frontal areas and hypermetabolism in orbitofrontal and parietal cortex and thalamus. Given the heterogeneity of findings, the authors concluded that the main pathophysiologic hypotheses of COVID-19–related hyposmia (i.e., olfactory cleft obliteration and neuroinvasion of SARS-CoV-2) do not explain dysosmia in all patients and that the PET findings probably reflect deafferentation and functional reorganization (29). In contrast, earlier case reports found orbitofrontal hypometabolism in patients with anosmia (30*,*^31^), which was interpreted as a result of direct neuroinvasion of SARS-CoV-2 via the olfactory bulb. As preliminary support for a possible extension of impairment to other brain structures, the latter groups also reported hypometabolism of medial temporal structures in a patient with COVID-19–related parosmia (32) and a patient without olfactory dysfunction (31) (in addition to other regions; Supplemental Table 1). A recent systematic study also found hints of an impairment of mesiotemporal structures in COVID-19–related olfactory dysfunction: Donegani et al. (33) prospectively recruited 14 patients with isolated hyposmia during the recovery phase from COVID-19 (4–12 wk after the first positive PCR result). Compared with control subjects and applying corrections for covariates (age, sex, scanner type) and multiple comparisons (i.e., *P* < 0.05, FWE-corrected), patients with isolated hyposmia were characterized by clusters of hypometabolism bilaterally in the parahippocampal and fusiform gyri and in the left insula, possibly reflecting cortical deafferentation. Beyond its implication in hyposmia, the involvement of limbic regions in COVID-19 may imply a risk of developing long-term neurologic (possibly cognitive) impairment, as discussed by the authors (33).

Other focal signs preliminarily investigated by ^18^F-FDG PET include a case of facial palsy with putative hypometabolism of the respective facial nerve (34) and a patient with frequent focal seizures possibly due to subacute encephalitis after SARS-CoV-2 infection with a normal ^18^F-FDG PET result (35) (Supplemental Table 1). Another group reported hypermetabolism of the inferior colliculi as a novel finding in patients with COVID-19 (*36,37*) that was associated with more frequent seizures and higher blood leukocytes at admission. However, the cause of this finding (e.g., inflammatory reaction (37), hyperactivation (36), and artifact) and its clinical relevance needs to be defined (Supplemental Tables 1 and 2).

### Encephalopathy

**FIGURE 1.**
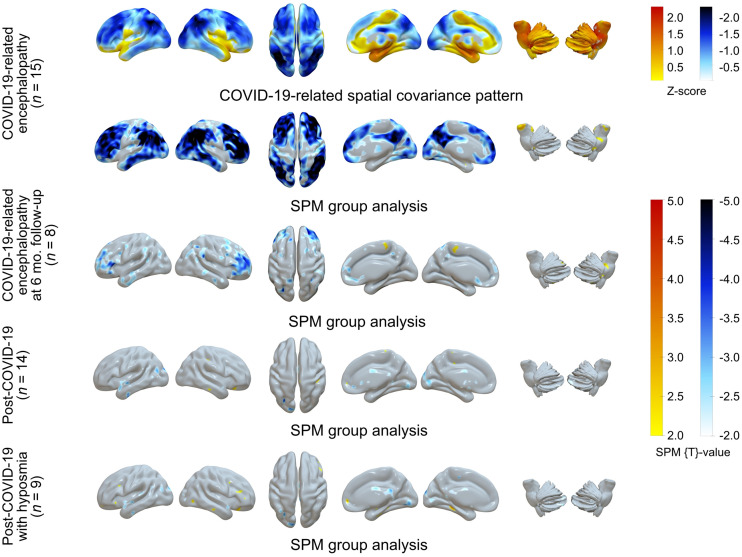
^18^F-FDG PET in COVID-19–related CNS disorders: principal-components analysis of spatial covariance pattern (first row) and SPM analysis of metabolic group differences (second to fifth rows) in patients with COVID-19–related encephalopathy (*n* = 15: first and second rows, Hosp et al. (18); of them *n* = 8 at 6-mo follow-up examination in third row, Blazhenets et al. (41)), patients with post–COVID-19 syndrome (*n* = 14; fourth row), and patients with post–COVID-19 syndrome and hyposmia (*n* = 9: fifth row, Dressing et al. (46)). Columns from left to right are lateral (left/right), superior, and mesial (right/left) views of cerebrum and lateral (left/right) views of brain stem and cerebellum (overlay created with Surf Ice 2 software; https://www.nitrc.org/projects/surfice/). Each analysis was performed in comparison to healthy controls (*n* = 13; 7 men and 6 women; mean age, 68 ± 7 y; PET performed under strictly comparable conditions), including age as covariate. SPM analyses entail count rate normalization to white matter (Hosp et al. (18); virtually identical results were gained with scaling to pons, whereas scaling to total brain parenchyma resulted in apparent [artificial] hypermetabolism in subcortical structures in COVID-19–related encephalopathy (40)). SPM results (*t* maps) were thresholded liberally for comprehensive display of findings (*t* = ±2, corresponding to *P* ≈ 0.05, uncorrected). Only extensive neocortical hypometabolism in COVID-19–related encephalopathy survives correction for multiple comparisons (voxel threshold, *P* < 0.05, false discovery rate–corrected).

All patients reported in this “Encephalopathy” section underwent inpatient and possibly intensive care unit treatment because of the overall high severity of COVID-19 symptoms. Furthermore, symptoms compatible with encephalopathy occurred with or briefly after the onset of general COVID-19 symptoms. Thus, the following study populations comprise a fairly homogeneous group. This homogeneity probably constitutes the basis for the observation that available ^18^F-FDG PET studies in COVID-19–associated encephalopathy yielded very consistent results (Table 1; Supplemental Table 1):

An initial case series by Delorme et al. ((38); *n* = 4) and subsequent systematic prospective studies by Kas et al. ((39); *n* = 7) and Hosp et al. ((18); *n* = 15/29 with PET, exhibiting symptoms compatible with encephalopathy) unanimously showed that the acute to subacute phase (±1 mo after infection) of COVID-19–associated encephalopathy is characterized by cognitive impairment (e.g., psychomotor agitation or slowing, executive functions, attention, visuoconstruction, and memory) and occasional other neurologic signs (e.g., hemiparesis, ataxia, apraxia, aphasia, myoclonus, and seizures). This clinical presentation is accompanied by a pronounced hypometabolism of frontoparietal-dominant neocortical areas (visual reads and conventional statistical parametric mapping [SPM] analyses, *P* < 0.05, FWE-corrected, or *P* < 0.01, false-discovery rate–corrected). Similar results were gained from a voxelwise, principal-components analysis–based comparison to age-matched controls, which yielded an extensive pattern of negative voxel weights (i.e., reduced metabolism) in frontoparietal-dominant neocortical areas and the caudate nucleus (18). Although an apparent hypermetabolism of striatum, pons, and cerebellar structures was found on individual visual reads and in statistically liberal (*P* < 0.05, uncorrected) group analyses (18*,*^38^*,*^39^), more thorough analyses suggest that these regions most likely show a preserved, actually not elevated, metabolism (18*,*^40^). Figure 1 shows the results of principal-components analysis and SPM group comparisons of patients with COVID-19–related encephalopathy compared with a group of healthy controls. Moreover, the individual expression score of the COVID-19–related spatial covariance pattern exhibited a highly significant, negative correlation with individual results from cognitive testing using the Montreal Cognitive Assessment (*r*^2^ = 0.62, *P* < 0.001 (*18*)).

Sequential follow-up data 1 mo later (39) and at 5–6 mo after infection (39*,*^41^) demonstrated a steady, though not necessarily complete, improvement of the clinical condition and ^18^F-FDG PET findings in most patients: in the study by Kas et al. (39), all but 1 patient showed a normal neurologic examination and recovered autonomy of daily living on follow-up. Still, all had abnormal cognitive evaluations with at least attentional or executive deficits, and 4 presented with psychiatric impairments. In parallel, cerebral metabolism improved on a group level, with mild residual hypometabolism of the left and right rectal gyri, the right insula, and the caudate nucleus and cerebellum. On a subject level, all but 1 patient, who got worse, showed a moderate to almost complete improvement (39).

Blazhenets et al. (41) provided the follow-up data of 8 patients from the COVID-19 register described by Hosp et al. (Table 1) (18): in parallel to a significant improvement in Montreal Cognitive Assessment performance (from 19 ± 5 to 23 ± 4, *P* = 0.03; still below normality [<26/30] in *n* = 5/8), follow-up ^18^F-FDG PET examinations showed a significant recovery (*P* < 0.01, false-discovery rate–corrected) of initial neocortical hypometabolism, with small areas of reduced metabolism being still detectable at a liberal statistical threshold (*P* < 0.005) at 6 mo. Likewise, the pattern expression score of the COVID-19–related spatial covariance pattern also significantly decreased over time (*P* < 0.005), albeit being still higher than in controls at a trend level (*P* = 0.06). Again, the pattern expression score correlated inversely with cognitive performance (repeated-measure *r*^2^ = 0.39, *P* < 0.01 (41)).

The authors of aforementioned studies agreed that COVID-19–related encephalopathy and the accompanying PET findings are unlikely to be caused by encephalitis due to neuroinvasion of SARS-CoV-2. The findings are more likely explained by a parainfectious systemic immune mechanism (e.g., cytokine release (18*,*^38^*,*^39^*,*^41^)), which according to an autopsy case in the study by Hosp et al. (18) manifests as pronounced microgliosis in the white matter, whereas the gray matter was mostly spared. Furthermore, it was suggested that an underlying neurodegenerative disease may be a predisposing factor (38), particularly in those who do not recover.

### Post–COVID-19 Syndrome

Post–COVID-19 syndrome is defined by persisting symptoms at 3 mo after symptom onset. Thus, it may include a highly variable group of patients in terms of initial disease severity, temporal course, and complaints. Patients still recovering from earlier manifestations (e.g., encephalopathy, usually treated as inpatients) may fall into this category, as will those who experience subjective complaints of brain fog only weeks after infection. Up to now, 1 preliminary case report (Supplemental Table 1) (42) and 4 systematic studies have been published (Table 2).

The team of Guedj et al. reported 2 studies on adults ((43); *n* = 35) and children ((44); *n* = 7) with persistent subjective complaints (mostly memory or cognitive complaints, fatigue, and insomnia) after apparent recovery from COVID-19. The mean or median delay between COVID-19 onset and PET was 3 and 5 mo in the adult and pediatric population, respectively. However, the time spans (26–155 d and 1–8 mo, respectively) indicate that both studies included subjects with ongoing COVID-19 according to the definitions (5) (they would also not fulfill the novel definition of post–COVID-19 condition by the WHO (20)). Both studies provided similar results (*P* < 0.05, FWE-corrected, in adults; *P* < 0.001, uncorrected, in children): compared with healthy adult subjects and control pediatric patients, long–COVID-19 patients showed extensive areas of hypometabolism including the orbitofrontal cortex bilaterally (in children at a liberal statistical threshold only), the medial temporal lobes bilaterally (hippocampus and amygdala; in adults right side only), the right thalamus (in adults only), and the brain stem and cerebellum. In adults, the number of complaints showed a weak negative association with brain stem and cerebellum uptake (*r*^2^ = 0.1 and 0.34, respectively). In their initial study on adults, the authors proposed neurotropism of SARS-CoV-2 through the olfactory bulb, with extension of impairment to the limbic and other mentioned regions (43), whereas they provided additional alternative explanations in the latter study on children (e.g., inflammatory, dysimmune or vascular changes, disturbance of gut–brain relationship, or psychologic causes (44)). Sollini et al. (45) conducted a case-control study enrolling 13 patients with symptoms (mostly dyspnea and fatigue; Table 2) persisting longer than 30 d after infection recovery (average, 4.4 mo) and extracted brain scans from whole-body ^18^F-FDG PET/CT examinations. Compared with surgically treated melanoma patients, patients with long–COVID-19 exhibited hypometabolism of the right parahippocampal gyrus and thalamus at a liberal statistical threshold (*P* < 0.001, uncorrected). Exploratory analyses (*P* < 0.005, uncorrected) linked symptoms such as anosmia, ageusia, or fatigue to additional regions such as the orbitofrontal cortex or brain stem (substantia nigra), respectively.

In contrast, Dressing et al. (46) recruited patients with neurocognitive symptoms persisting for more than 3 mo after infection (6.6 mo on average; retrospectively, all meeting the criteria of the post–COVID-19 condition by the WHO (20)). All patients complained of attention and memory problems, most also complained of fatigue, and 39% could not return to previous levels of independence or employment. However, on extensive neuropsychologic testing, half the patients were completely unimpaired, whereas other patients showed mild to moderate impairment in single domains (most frequently visual memory, 21% of cases). The most consistent finding was fatigue (61%; cognitive fatigue, 67%) when using a self-rating questionnaire. Fourteen patients underwent ^18^F-FDG PET that yielded no significant regional abnormality in comparison to control patients on either a single-subject or group level (using both SPM [*P* < 0.005, uncorrected] and principal-components analysis; Fig. 1). No correlation between clinical variables and PET measures could be established. Given the striking discrepancy between these findings and findings in COVID-19–related encephalopathy, the authors proposed that mechanisms other than those in encephalopathy probably contribute to post–COVID-19 syndrome, fatigue being foremost (46).

## CRITICAL APPRAISAL OF PET AND SPECT LITERATURE

According to the WHO (https://covid19.who.int/), there had been almost 250 million confirmed COVID-19 cases and 5 million deaths by November 1, 2021. In parallel, an unprecedented wealth (or flood) of scientific reports on COVID-19 was published, amounting to 92,940 MEDLINE entries in 2020 and 114,360 in 2021 up to November 1 (search term “COVID-19”; >300 reports per day since January 1, 2020). Although there is no doubt that the COVID-19 pandemic required a fast and collective response of medical research, it is also a frequent perception that in the early phase of the pandemic nearly everything labeled with the term “COVID-19” got published quickly, sometimes too quickly. Still, many questions await definite answers (e.g., mechanism underlying COVID-19–related CNS changes), and it is worthwhile critically gauging existing reports. For instance, a note of caution has recently been published regarding the quality of mental health research in the COVID-19 pandemic, raising concerns about the validity, generalizability, and reproducibility of findings (47).

There are several recurring issues, some of which will be briefly discussed in the following sections. They should be kept in mind when considering the present results. They also represent a request and motivation for further studies.

### Study Design and COVID-19 Populations

Given the still high incidence of COVID-19, there is no necessity to rely on retrospective analysis of convenient samples. They pose an inherent risk of bias and, because of the lack or inconsistency of data, do not allow for in-depth analyses of clinicoimaging correlations. Likewise, case reports or series are hardly justified except for very rare conditions, for which they may constitute an interesting starting point. In addition, it is evident that currently available longitudinal studies provided the deepest insight into underlying mechanisms and their course (39*,*^41^).

The latter studies demonstrated a strong time dependency of COVID-19–related CNS changes, underlining the need to clearly define the time window of inclusion of patients with respect to the time of SARS-CoV-2 infection. Likewise, it is apparent that a large fraction of inpatients has COVID-19 encephalopathy (∼70% (18)), which takes several months to recover from (39*,*^41^*,*^48^). Despite objective cognitive impairment in these patients, subjective perception of deficits and psychologic strain is frequently lacking in this group (49). In contrast, symptoms such as stress, anxiety, and reduced wellbeing are more prevalent after a mild course of disease (49). Accordingly, long-term symptoms such as subjective cognitive impairment and fatigue typically occur in patients who are younger (<50 y) and have a low rate of hospitalization (<30%; (2*,*^19^)). As the definition of “post–COVID-19 syndrome” used so far reflects only temporal aspects (5), there is a tremendous risk of building mixed populations in terms of initial disease severity, treatment, and impairment (subjective/objective). Proper selection of patients will be key in separating protracted COVID-19–related encephalopathy from the post–COVID-19 syndrome in a strict sense (2*,*^19^). In this respect, the novel, stricter WHO definition is welcome progress (20). In fact, available PET studies do not support the idea that neurocognitive post–COVID-19 syndrome represents a protracted manifestation of COVID-19–related encephalopathy, because available studies on long–COVID-19 or post–COVID-19 syndrome either yielded no metabolic alteration at all (46) or depicted metabolic changes (43–45) that do not match the highly consistent findings in COVID-19–related encephalopathy (18*,*^39^). Concerning the diagnosis of COVID-19, a PCR or at least state-of-the-art antibody proof should be mandatory. From a clinical perspective, typical clinical findings may be sufficient to ground a diagnosis in a pandemic situation, but this is not sufficient for a scientific study trying to unravel a specific disease.

Up to now, none of the reviewed studies has conducted a proper sample size calculation. Likewise, study registration appears to be an exception (e.g., (18*,*^46^)) that needs to be turned into a rule.

### Control Populations

The use of convenient samples seems to be an even bigger problem when it comes to PET reference datasets. In fact, the descriptions of methods of most studies do not clearly state whether reference populations were indeed examined under comparable circumstances (e.g., identical scanners, acquisition techniques, reconstruction techniques, or patient preparation). The contribution of technical differences may be negligible if disease effects are large but may be a crucially biasing factor if effects are small (e.g., post–COVID-19 syndrome). Some authors acknowledge inconsistencies and conducted confirmatory analyses (18), whereas most authors ignored this issue. The use of oncologic control patients may be particularly problematic if brain scans are cropped out of whole-body scans, which are usually acquired and reconstructed differently from brain scans. Furthermore, preparation of brain scans requires well-defined resting (or neutral) conditions, whereas adherence to these conditions is lower in patients undergoing whole-body PET/CT (patients may read, talk, or use their smartphones).

Since the publication of the original studies by our group relying on control patients (i.e., patients with unclear neurologic or psychiatric complaints, in whom a somatic CNS disease was carefully excluded (18*,*^41^*,*^46^)), we had the opportunity to scan a cohort of thoroughly evaluated healthy controls. The local ethics commission and federal office for radiation protection approved this study, and all subjects gave written informed consent. Using these subjects as a reference cohort, we were able to confirm our initial results (Fig. 1). We also conducted an additional analysis on subjects with post–COVID-19 syndrome and hyposmia, but we found no significant hypometabolism of the olfactory regions as described by other groups.

### PET Data Analysis

Comparison of datasets by common statistical methods such as SPM requires count rate or intensity normalization. It is well known that extensive, disease-related metabolic alterations in one direction may cause artificial alterations in the other direction on conventional analyses, if diseased brain regions are part of those used for normalization. In cases of neocortical hypometabolism and normalization by whole-brain proportional scaling, apparently “hypermetabolic” areas often include the basal ganglia, thalamus, mesiotemporal regions, brain stem, and cerebellum (40). Several lines of evidence such as ROI analyses (plasma glucose–corrected uptake values), clinicoimaging correlations, and follow-up studies suggest that the areas of hypometabolism in subacute settings most likely reflect true hypometabolism (40). However, in our opinion, and with the possible exception of concomitant autoimmune encephalitis, it is still questionable whether regions of hypermetabolism reported in several studies exhibit true hypermetabolism or inflammation. On one hand, the latter is not convincingly supported by histopathologic data. On the other, an artificial increase seems much more likely, especially if global count rate normalization was applied (40). Still, given the systemic activation of microglia and astrocytes (albeit being most pronounced in the brain stem and cerebellum), one cannot exclude that associated metabolic changes cause some bias if white matter is used as a reference region (18*,*^41^). One way to avoid this problem may be to conduct principal-components analyses as proposed by Hosp et al. (18).

Given the complexity, costs, and radiation exposure, cohorts in PET studies are often rather small, which limits their statistical power. Thus, a pragmatic approach may be to define a liberal statistical threshold and avoid a correction for multiple comparisons. This may lead to type I errors. Several systematic studies (Tables 1 and 2; Supplemental Table 2) used liberal thresholds; consequently, this limitation needs to be kept in mind when considering anatomically (e.g., small clusters near or within CSF spaces (45)) or functionally (e.g., weak correlations with subjective, ill-defined complaints (43)) questionable findings. Such limitations particularly apply to case reports or smaller series, which often suffer from additional flaws (e.g., reliance on observer-dependent visual reads, lack of coregistration to high-resolution structural imaging or MRI; Supplemental Table 1).

### Clinicoimaging Correlations

Whereas clinicoimaging correlations cannot prove causality, they may be useful to check for plausibility. For instance, the high correlation of frontoparietal-dominant changes in cerebral metabolism and formally assessed cognitive functions served by frontoparietal regions in COVID-19 encephalopathy (18) supports the notion that these changes depend on each other. The magnitude of metabolic alterations was also in line with imaging studies on patients with comparable cognitive deficits (e.g., dementia).

In turn, some imaging findings need further elucidation: for instance, bilateral hyperactivation of the inferior colliculi (36*,*^37^) may be expected to interfere with normal auditory functioning, which, however, was not reported. Given that this finding was reported in rather severely affected patients, one may also consider alternative explanations (e.g., artificial, relative hypermetabolism due to neocortical hypometabolism). Donegani et al. (33) reported a bilateral hypometabolism of the parahippocampal and fusiform gyri in isolated hyposmia. Although they used an 8-item odor test, no correlation analyses were reported. In turn, one may also have expected other deficits such as memory impairment, which unfortunately was not addressed. In cases of post–COVID-19 syndrome, the situation is even more complex: patients commonly complain about multiple symptoms with a high impact on daily functioning, which, however, cannot be verified in such magnitude by formal testing (46*,*^49^). Still, extensive patterns of hypometabolism have been reported (43*,*^44^), which on first glance may be expected to result in various additional symptoms (e.g., severe objective amnestic problems). Unfortunately, no formal testing was pursued. In contrast, no such metabolic changes were observed in the study by Dressing et al. (46), underscoring the need to verify symptoms and complaints by operationalized formal assessments to gain deeper insights and unravel discrepancies. Such assessments will also benefit from inclusion of additional correlative biomarkers (e.g., PET or CSF biomarkers of neurodegeneration) and, ultimately, imaging–neuropathology correlations, which are still very sparse (18).

### Correlations with Knowledge from Related Disciplines

Imaging findings and concepts derived from them have to be aligned with knowledge from related disciplines. It is evident that neurologic or neurocognitive findings in COVID-19 cover a broad spectrum of disciplines. Neuropathologic findings and psychiatric concepts may be considered as 2 distant ends of such a spectrum. However, we believe that these disciplines are particularly helpful for a better understanding of current findings.

#### Insights from Neuropathologic Studies

Whereas an early neuropathologic investigation of patients who died from COVID-19 found primarily hypoxic damage (50), more recent reports draw a vivid picture on how COVID-19 may affect the CNS (51*,*^52^). Major findings include an activation of microglia (formation of microglia nodules in some patients) and astrocytes, which was pronounced in the brain stem and cerebellum and affected white rather than gray matter. Detection of SARS-CoV-2 (spike protein or RNA) succeeded in approximately 50% of patients, especially in the brain stem and cranial nerves. This detection is remarkable, as the neuroinvasive potential of β-coronaviruses has been broadly discussed (e.g., after nasal inoculation, with axonal and transsynaptic propagation) (53*,*^54^). However, because of its sparse detection rate in neuropathologic samples and the absence of lytic figures, infected neurons, or glial cells (51), proofs for direct neuroinvasivity and the occurrence of neuron-to-neuron propagation within the human CNS are lacking. Regarding adaptive immunity, infiltration of CD8 and CD4 T cells especially occurred in the perivascular compartment, being associated with blood–brain barrier dysfunction. Interestingly, the immune activation within the perivascular compartment is specific for COVID-19 and differed from findings in patients with multiple sclerosis or non–COVID-19 respiratory failure (52). Although the SARS-CoV-2 spike protein has been occasionally detected in perivascular cells (52), neuroinflammatory changes are more likely induced by circulating cytokines and inflammatory mediators in the course of a systemic inflammatory response (51*,*^55^). These data were derived from unselected patient populations, as the presence of neurologic symptoms was not registered. Thus, additional studies with detailed clinicopathologic correlations are warranted. In addition, PET imaging of activated microglia might represent a promising imaging technique in COVID-19 but to the best of our knowledge has not been explored yet.

#### Current Concepts of Fatigue

Fatigue might be translated as “extreme and persistent tiredness, weakness or exhaustion that could be mental, physical or both” (56), but there is no further agreement on the definition of this entity (57). Fatigue can cooccur with clinical symptoms such as depression, pain, sleep impairment, and cognitive dysfunction (58) and can be associated with chronic medical conditions (e.g., postviral infection, cancer, inflammatory bowel disease, multiple sclerosis, depression, and fibromyalgia (59)). Thus, a multifactorial etiology with strong impact of somatic as well as psychosocial risk factors has been postulated (60). Fatigue has a significant symptom overlap and comorbidity with psychiatric disorders (61). This is in line with a study investigating predictors of fatigue in individuals recovering from the acute phase of COVID-19: while the female sex and a comorbidity of depression or anxiety were identified as significant predictors for developing fatigue, indicators of COVID-19 severity, markers of peripheral immune activation, and circulating proinflammatory cytokines did not reach statistical significance (62). Still, fatigue seems to be distinct from psychiatric disorders; however, perceptions, attributions, and coping skills of patients with psychiatric comorbidity may perpetuate the fatigue condition (61). This notion is reinforced by a significant therapeutic response of fatigue to cognitive behavior therapy (63). As far as permissible and based on the rather disappointing experience with ^18^F-FDG PET in psychiatric and somatoform disorders, one may expect that the contribution of ^18^F-FDG PET to understanding and diagnosing a post–COVID-19 syndrome dominated by fatigue is actually limited.

## PERSONAL PERSPECTIVE: USE OF MOLECULAR IMAGING IN COVID-19–RELATED CNS DISORDERS

From a scientific point of view, molecular imaging of COVID-19–related CNS disorders represents an exciting field that not only contributes to the understanding of disease mechanisms in COVID-19 but may also provide important implications for other conditions (e.g., neurodegeneration, encephalopathies, and delirium). In this regard, we believe that carefully designed studies on COVID-19 populations are of great interest.

Concerning the clinical application of molecular imaging for diagnosis and management of COVID-19–related CNS disorders, we actually see little impact and, consequently, little need to adjust current clinical practice: ^18^F-FDG PET is gaining increasing acceptance for differential diagnosis and follow-up of encephalitis, often showing superior performance over MRI (64*,*^65^). Even more so, PET and SPECT are established methods for differential diagnosis of neurodegenerative CNS disorders such as parkinsonism (66*,*^67^). Accordingly, in patients with suspected encephalitis or parkinsonism in the context of COVID-19, PET and SPECT may be performed according to established clinical pathways. Follow-up studies would in fact be of particular interest for differentiating between possible reversible COVID-associated nigrostriatal dysfunction and progressive, presumably COVID-independent neurodegenerative parkinsonism. Molecular imaging is also accepted for differential diagnosis in cognitive impairment and dementia (68). However, in the context of COVID-19, it is probably advisable to define the need for additional imaging studies based on the clinical presentation and time course. In cases of possible COVID-19 encephalopathy, a reversible cortical dysfunction can be assumed, while in post–COVID-19 syndrome with predominant fatigue and without decisive pathologic findings on neuropsychologic testing, a pathologic imaging correlate is actually unlikely. In our opinion, patients with “subjective” neuropsychiatric deficits as potential sequelae of COVID-19 should undergo a comprehensive clinical work-up including validated neuropsychologic and neuropsychiatric testing to verify deficits. A diagnostic benefit from ^18^F-FDG PET may be expected only if a cognitive impairment is verified and persisting (>3–6 mo) or progressive.

## DISCLOSURE

No potential conflict of interest relevant to this article was reported.
